# Diagnostic challenges in laryngeal lymphoepithelial carcinoma: A case report of misdiagnosis and clinical implications

**DOI:** 10.1016/j.ijscr.2025.112015

**Published:** 2025-10-10

**Authors:** Huji Zhang, Zhongjiao Chen, Lina Wang, Long Wang

**Affiliations:** Guiqian International Hospital, Guiyang City, Guizhou Province, China

**Keywords:** Laryngeal, Lymphoepithelial carcinoma, Schmincke pattern, CD20, Case report

## Abstract

**Introduction and importance:**

Laryngeal lymphoepithelial carcinoma (LEC) is a rare and diagnostically complex malignancy, often overlooked due to its nonspecific presentation and common association with smoking. Prompt and accurate diagnosis is crucial for achieving optimal patient outcomes.

**Case presentation:**

A 63-year-old Chinese male with a two-year history of persistent hoarseness and throat discomfort initially diagnosed as chronic inflammation. However, further evaluation revealed vestibular fold swelling and right vocal cord thickening. Surgical resection confirmed laryngeal LEC with a Schmincke pattern, CD20+ B-cell infiltration, positive cytokeratin (CK) and p40 immunostaining, and negative Epstein-Barr virus-encoded RNA (EBER). Following radiotherapy, the patient experienced symptomatic improvement.

**Clinical discussion:**

Laryngeal LEC is often EBER-negative and challenging to diagnose. This case highlights the need to consider LEC even with benign-appearing findings. Accurate diagnosis relies on detailed histology and IHC, especially CK and p40.Radiotherapy is an effective treatment for EBER-negative laryngeal LEC.

**Conclusion:**

Laryngeal LEC is a smoking-associated, EBER-negative rare malignancy that typically responds well to radiotherapy, yielding a favorable prognosis. This case underscores the necessity of considering laryngeal LEC in similar clinical presentations to avoid misdiagnosis.

## Highlights

• Rare case of LEC initially misdiagnosed as chronic inflammation
• Schmincke pattern and B-lymphocyte-rich stroma complicated intraoperative frozen section diagnosis
• Highlights the importance of considering laryngeal LEC in differential diagnosis of laryngeal lesions
• Emphasizes the role of IHC (CK, p40, p53) and FISH (EBER) in accurate diagnosis of laryngeal LEC
• Reports successful treatment with radiotherapy, underscoring favorable prognosis of laryngeal LEC

## 1 Introduction

Lymphoepithelial carcinoma (LEC) is a malignant tumor characterized by undifferentiated, non-keratinizing epithelial cells and a stroma rich in lymphoid tissue [1]. The most common site for LEC occurrence is the nasopharynx within the head and neck region. Unlike nasopharyngeal LEC, which is associated with Epstein-Barr virus (EBV) infection, laryngeal LEC is often closely linked to smoking. Due to the absence of specific clinical manifestations, laryngeal LEC is frequently misdiagnosed as chronic inflammation or other benign lesions. This article reports a case of a male patient with laryngeal LEC who was misdiagnosed during intraoperative frozen pathology and discusses its related clinical and pathological features.

## 2 Case presentation

The patient is a 63-year-old Chinese male who began experiencing hoarseness without an obvious cause two years ago. He frequently felt a foreign body sensation in the throat, accompanied by difficulty speaking, with persistent symptoms that did not improve with rest; however, he reported no difficulties with swallowing or other discomforts. The patient had previously visited multiple hospitals and underwent laryngoscopy and mucosal tissue biopsies, all of which were diagnosed as chronic inflammatory changes. Seeking further diagnosis and treatment, he came to our hospital.

**Fig. 1:**
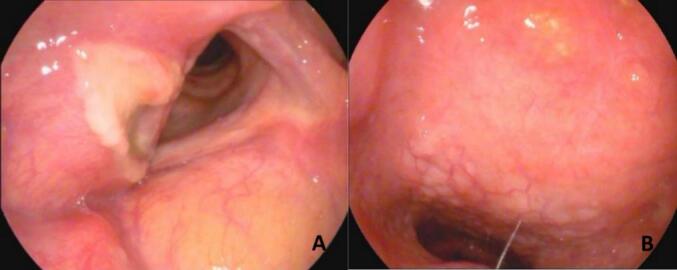
(A) Surface mucosal erosion was observed on the right side of the vestibular fold; (B) the middle and posterior sections of the right vocal cord were slightly elevated, displaying surface vascular engorgement, while the mucosal surface appeared smooth.

Upon initial examination at our hospital, an electronic fiber laryngoscopy revealed swelling on the right side of the vestibular fold, with surface mucosal congestion and erosion (Fig. 1A), partial obstruction of the glottis, and poor glottic closure. The middle and posterior sections of the right vocal cord were slightly elevated, exhibiting surface vascular engorgement while the mucosal surface appeared smooth (Fig. 1B). CT scan results showed irregular thickening of the right vocal cord and ventricular fold, characterized by an irregular soft tissue density lesion measuring approximately 10 mm × 20 mm, with unclear margins and uniform density. Contrast scans indicated mild uniform enhancement, resulting in narrow compression of the laryngeal ventricle, leading to a radiological diagnosis highly suggestive of a benign space-occupying lesion, with a strong likelihood of inflammatory changes. The patient had a 40-year smoking history, along with a 6-year history of diabetes and a 20-year history of hypertension. Other systemic examinations revealed no abnormalities.

**Fig. 2:**
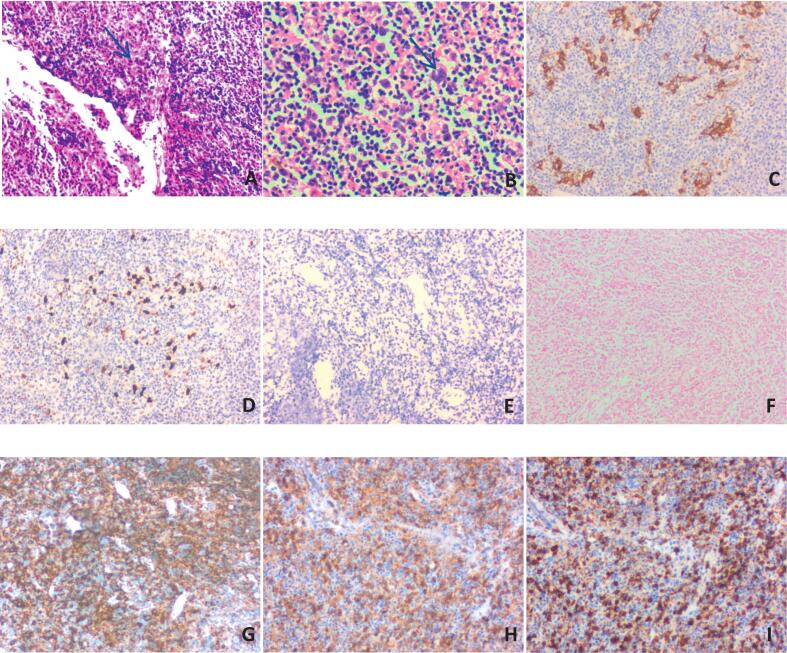
(A) Sporadic atypical large cells were distributed in the mucosal tissue in frozen section; (B) tumor cells exhibited the Schmincke pattern, characterized by their arrangement as single cell or small clusters; (C) IHC was positive for CK; (D) p53 association with >80 % mutated cells; (E) IHC was negative for P16; (F) FISH for EBER was negative; (G) A rich population of CD20+ B lymphocytes surrounded the tumor cell clusters; (H) CD4+ T cells surrounding the tumor cell clusters were sparsely distributed; (I) CD8+ T cells surrounding the tumor cell clusters were also distributed sparsely.

To further clarify the diagnosis and to explore treatment options aimed at symptom relief, the patient opted for surgical resection and biopsy of the occupying lesion. During the procedure, a small section of mucosal tissue was excised via microscopic-assisted laser laryngoscopic resection for examination. Gross observation revealed two pieces of grayish-white mucosal tissue measuring approximately 10 mm × 8 mm × 2 mm. Frozen section revealed ulcerated areas with granulation tissue formation on the mucosal surface, extensive infiltration of inflammatory cells, and sporadic atypical large cells(Fig. 2A). The intraoperative pathological diagnosis indicated that the mucosal tissue exhibited chronic inflammatory changes.

Postoperatively, routine histopathological examination revealed scattered clusters of atypical cells within the mucosa. These atypical cells exhibited unclear cell membranes, sparse cytoplasm, enlarged nuclei with irregular nuclear contours, and prominent nucleoli(Fig. 2B). Immunohistochemical (IHC) demonstrated that the atypical cell clusters were positive for CK (Fig. 2C)and P40 expression, as well as exhibiting mutant pattern expression of p53(Fig. 2D), while negative for p16 (Fig. 2E). In situ hybridization (FISH) for EBER (Fig. 2F) yielded negative results. Additionally, the lymphoid tissue surrounding the atypical cell clusters (Fig. 2G) contained abundant CD20-positive B lymphocytes (Fig. 2G) and T lymphocytes (Fig. 2H, I), which were uniformly intermingled. The final pathological diagnosis confirmed. Laryngeal LEC, with the tumor cells being EBER-negative. Following a discussion with the clinical team, the patient opted for radiotherapy (total dose of 50–54 Gy: conventionally fractionated at 2 Gy/fraction, 5 fractions/week, for a total course of approximately 6 weeks) instead of further surgical intervention. After three months of radiotherapy, the patient returned for a follow-up visit and reported an improvement in his clinical symptoms.

### 2.1 Patient's perspective

The patient reported significant relief after diagnosis and radiotherapy. He described experiencing two years of anxiety due to persistent hoarseness and inconclusive diagnoses. He expressed gratitude for the hospital's thorough care and clear communication. After the cancer diagnosis came out, he felt very comforted because he had a good radiotherapy treatment plan.

## 3 Methods

This case report has been prepared and reported in accordance with the SCARE criteria [12].

## 4 Discussion

Laryngeal LEC is a rare subtype of poorly differentiated squamous cell carcinoma, characterized by significant reactive lymphocytic infiltration and exhibiting morphological features similar to those of nasopharyngeal LEC. To date, there have been fewer than 50 documented cases confirmed through IHC and FISH for EBV [[1], [2], [3]]. Unlike nasopharyngeal LEC associated with EBV infection among Chinese males commonly, laryngeal LEC predominantly presents in elderly Caucasian males and is closely associated with smoking.

The clinical manifestations of laryngeal LEC are non-specific, typically presenting as dysphagia, hoarseness, and odynophagia—symptoms which often overlap with those of ordinary laryngitis and conventional squamous cell carcinoma [4]. Currently, laryngoscopy serves as a critical screening tool for laryngeal cancer and enables definitive diagnosis through biopsy. Typical findings during laryngoscopy for laryngeal LEC include irregular masses or protrusions on the vocal cords, which may be significantly thickened, resulting in restricted movement of the right vocal cord and incomplete glottic closure [5]. However, these non-specific symptoms complicate differentiation from conventional squamous cell carcinoma. In the present case, multiple prior laryngoscopies conducted at external facilities suggested a high likelihood of inflammatory lesions, with biopsy results consistently indicating chronic inflammation. A repeat review of the previous external biopsy slides at our institution revealed no evidence of malignant cells. This misdiagnosis, or delayed diagnosis, may be attributed to the complex anatomical structure of the larynx and the proficiency of the examining clinician, particularly in cases involving early endophytic lesions where improper sampling can lead to false-negative results and subsequent delays in treatment.

The diagnosis of laryngeal LEC primarily relies on biopsy pathology and IHC. Histologically, the tumors exhibit morphological characteristics akin to those of nasopharyngeal LEC, and they can be classified into two distinct patterns: the Regaud and Schmincke patterns, with the Regaud pattern being the more prevalent [6]. Tumor cells exhibiting the Regaud pattern typically demonstrate sheet-like or nested arrangements characterized by large pleomorphic nuclei and prominent nucleoli. In contrast, the Schmincke pattern is characterized by fewer tumor cells arranged as single cells or small clusters. In this case, the tumor cells displayed a scattered distribution within the mucosal tissue consistent with the Schmincke pattern and were surrounded by an abundance of lymphocytes. The Schmincke pattern distribution complicated the identification of tumor cells during intraoperative frozen section analysis, leading to an initial misdiagnosis. Despite the review of the frozen sections by five experienced pathologists postoperatively, a definitive diagnosis of malignancy was not established, and tumor cells were not identified by one pathologist during the routine postoperative examination.

IHC and FISH are important methods for diagnosing laryngeal LEC. Tumor cells characteristically exhibit positivity for CK and squamous markers such as p40 and P63, alongside a mutation-type expression of p53. The result of FISH for EBER is typically negative, which distinguishes laryngeal LEC from metastatic carcinoma. Given that the stroma of laryngeal LEC is rich in lymphoid tissue, it is crucial to differentiate it from lymphoma and chronic mucosal inflammation. Key points for differentiation from chronic inflammatory lesions include the positive expression of CKs and p40 within tumor cells. In contrast, differentiation from lymphoma requires assessing the negative expression of CD45, CD3, and CD20 by tumor cells, as well as analyzing the surrounding lymphocyte distribution pattern. Of note, this case presented a rarely described finding: an enrichment of B lymphocytes surrounding the tumor tissue, contrasting with the typical LEC microenvironment, which is often characterized by a dominance of T lymphocytes as described in existing literature [7]. This phenomenon has not been previously reported in the literature. These observations suggest that the Schmincke pattern, combined with the unique tumor microenvironment observed in this specific case, could potentially represent early pathological features. However, further research is necessary to better understand this phenomenon, especially given the current lack of large-scale specimen analyses.

Given the rarity of laryngeal LEC, there is currently no global consensus regarding its diagnosis and treatment [1,8]. The therapeutic approach typically involves a multimodal strategy, including surgical resection, radiotherapy, and chemotherapy. Emerging research suggests that laryngeal LEC is highly responsive to radiotherapy, achieving favorable local control rates; most patients experience positive outcomes following treatment [9]. For patients with advanced laryngeal LEC, surgery is generally recommended to reduce tumor burden, followed by radiotherapy [10,11]. In this case, due to mild symptoms and the absence of significant tumor mass or structural compromise observed on preoperative CT, the patient underwent radiotherapy. At the three-month follow-up, the patient reported an improvement in symptoms and overall health status.

In conclusion, laryngeal LEC is a rare malignancy typically exhibiting negative EBER expression, and is associated with risk factors such as smoking. For this condition, early and accurate pathological diagnosis, combined with a well-considered treatment strategy, are crucial for significantly enhancing patient outcomes and overall health status. The misinterpretation of intraoperative findings in our case underscores the critical importance of considering laryngeal LEC within the differential diagnosis in similar clinical presentations to prevent misdiagnosis. This heightened awareness is vital for both general otolaryngologists and pathologists, enabling them to avoid diagnostic pitfalls and ultimately improve patient care and well-being.

## Abbreviations

LEC: Lymphoepithelial carcinoma
EBV: Epstein-Barr virus
IHC: Immunohistochemistry
ISH: In situ hybridization

## CRediT authorship contribution statement

Huji Zhang: Involved in data curation, manuscript conception, and drafting of the manuscript.
Lina Wang: Pathological diagnosis.
Zhongjiao Chen: Performed immunohistochemical and hematoxylin-eosin staining.
Long Wang: Contributed to pathological diagnosis, manuscript conception, manuscript revision, guarantor.

## Informed consent statement

Written informed consent was obtained from the patient for publication of this case report and any accompanying images. A copy of the written consent is available for review by the Editor-in-Chief of this journal on request.

## Ethical approval

This study was approved by the Ethics Committee of Guiqian International Hospital (GQYY-LC-202508180080). The date of approval by the ethics committee is August 18, 2025. Our institution approved the publication of the case details.

## Guarantor

Long Wang accepts full responsibility for the integrity of the work and confirms that he had full access to all the data and controlled the decision to publish.

## Funding

N/A.

## Declaration of competing interest

N/A.

## Data Availability

All data are included in this article.
